# Estimation method for distance cost to access medical services: Policy and patient privacy implications in Taiwan

**DOI:** 10.3389/fpubh.2022.1065742

**Published:** 2022-12-22

**Authors:** Siao-Jing Guo, Hsing-Chu Chen, Chia-Feng Yen

**Affiliations:** ^1^Head Office, Hualien Tzu Chi Hospital, Buddhist Tzu Chi Medical Foundation, Hualien, Taiwan; ^2^Department of Public Health, Tzu Chi University, Hualien, Taiwan

**Keywords:** distance cost, medical service access, patient privacy, QGIS resource allocation distance cost, QGIS resource allocation

## Abstract

**Introduction:**

Indicators of healthcare access with high reliability, validity, timeliness, and easy application can aid in an understanding of the supply and demand of a region's medical resources and assist governments in allocating resources more effectively. However, a key concern when developing indicators is the protection of private information, such as patients' residential addresses.

**Objectives:**

We develop an estimation method for distance cost using official public information, including a region's disease prevalence rates and population.

**Materials and methods:**

The method accounts for patients' privacy and addresses limitations associated with using the National Health Insurance Database. This cross-sectional study conducts a secondary data analysis using SPSS and QGIS. The data were divided into a validation group and an index development group with the medical distance calculated for each group. Data for the validation group were sourced from the medical records of patients with diabetes (*n* = 108–164) and hypertension (*n* = 243–348) in Yuli documented by a medical center in 2017–2019, and the data for the novel index development group included diabetes and hypertension prevalence sourced from national official public data. The study compared the consistency of the two groups' medical treatment distances to verify the accuracy of the estimation method.

**Results:**

The estimated distances for the index development group showed a high consistency (ICC > 0.9). Further, the index development group had an excellent R-square after adjusting for age (98.1%) and gender (92.7%).

**Conclusions:**

The proposed method to estimate healthcare on the basis of disease prevalence and population protects patient privacy and can be implemented by local governments.

**Trial registration:**

This study was approved by the Research Ethics Committee of the Hualien Tzu Chi Hospital, Buddhist Tzu Chi Medical Foundation (IRB109-239-B).

## Introduction

Taiwan's National Health Insurance system, implemented in 1995, has not only eased financial and economic barriers impeding healthcare access but has also contributed toward alleviating poverty resulting from illness. However, numerous studies have highlighted inadequate or unequal medical resources in Taiwan's remote areas despite the health insurance system (1–3). The Taiwanese government has been making efforts to gradually improve the uneven distribution and inconsistent quality of medical care through the regionalization of healthcare since 1985. Yet, there are still serious concerns about the insufficient and unequal distribution of medical care resources in rural areas and the long distance between doctors and those seeking medical care.

Decision makers for healthcare policies largely adopt the number of specialist medical personnel, the number of hospital beds and the ratio of medical services to a region's population as indicators of medical care access, none of which require geographical information (4–9). Other accessibility indicators include the distribution density of medical resources, distance to medical institutions, travel duration and individual medical care needs. These indicators require patients to provide geographical and private information, which are used for parametric inferential statistics. These indicators relate to the more critical issue of the justice and equity of resource allocation (3, 10, 11).

Studies have shown that distance impacts the utilization and demand for medical services, as when the distance between consumers and medical providers or the time required to access medical care are greater, medical utilization and demand are lower (12, 13). Therefore, the accurate measurement and appropriate configuration of regional medical care services are necessary to effectively improve healthcare services.

Actual medical distance can be measured on the basis of patients' residence and terrain or transportation route from residential location to a medical institution. In addition, it is necessary to account for other factors influencing access to medical resources in a region such as time taken to receive treatment, particularly during rush hour.

The indicators of the two-step floating catchment area (2SFCA) and enhanced 2SFCA (E2SFCA), developed between 2005 and 2016, require spatial information as well as patients' personal data such as age, gender, race, income, occupation and urban development information of patients' residence, thus rendering them tedious to implement (14–17). Lin et al. (2) and Yen and Lin (3) introduced significantly simplified indicators, although they also used the distance between the patient residence and healthcare provider. Overall, 2SFCA and E2SFCA were developed by many variables which are usually privacy information. They are non-accessible and have no immediacy for health policy. The indexes developed by Yen and Lin et al. are briefer, but still, need the address of the patient which is also private data. So in the decision maker's view, streamlined, instant, and accurate distance cost metrics are very important. In sum, where the patient lives is critical for accurately estimating the distance cost (2, 3).

Developed democracies have implemented privacy laws to protect personal information as a fundamental human right. Taiwan's National Health Insurance Database has a 99% coverage rate and contains medical records and conditions, thus making it an important resource when estimating the utilization of and demand for healthcare services. Taiwan's Personal Data Protection Act (18) applies across both the public and private sectors. The National Health Insurance Database lacks social factors and documents a patient's address only at the regional level. In the past, the database was used to estimate the medical distance between hospitals and administrative regions, such as towns. However, for Hualian County, the estimation of distance between users and a medical caregiver is limited to the county's 13 towns. Such over-simplified and biased information impacts the accurate estimation of distance cost for medical care, especially for remote areas where residents are highly dispersed. Hualien county, surrounded by mountains terrain and only 10% narrow range plain, with a total area of 4,628 square kilometers is the largest county in Taiwan. Compare to the capital in Taipei city has 9 medical centers, Hualien county has only one (Hualien Tzu Chi Hospital). According to the Central Health Insurance Agency of the Ministry of Health and Welfare, 7 towns out of 13 in Hualien County are included in the “77 Areas Lacking Medical Resources of the National Health Insurance in 2022” (19). Distance barriers are one of the factors contributing to health inequalities, and accurate and immediate calculation of distance costs will better reflect government subsidy needs than population numbers.

Thus, there is a need to develop a method that accurately estimates the cost of the distance between users and medical care providers and that uses more valid indicators than a patient's Insured unit registered by health insurance at the administrative or “township” level and that does not require the patient's exact address. Given that the population is not uniform within a township, but rather is clustered and randomly distributed, using a weighted center of population, which combines populations and geographical centers, allows for a more accurate calculation of travel distance (20), especially in remote regions with uneven population density. The proposed method will also protect patient privacy and address the limitations of various databases that use location variables at the townships level.

Diabetes and hypertension are highly prevalent chronic diseases in eastern Taiwan. This study focuses on patients with diabetes and hypertension, as these conditions have high rates of serious complications, including cardiovascular disease, stroke, skin ulceration, retinopathy, neuropathy, kidney failure and amputation (21). In 2019, diabetes was the fifth leading cause of death in eastern Taiwan, and about 50% of all diabetic patients die from heart disease and stroke. Referencing official data on the prevalence of the diseases and area populations as an index development group, we used the number of cases and their distance to medical treatment as key variables when developing the estimation method and used real outpatient data from a medical center to calculate the actual medical distance for a verification group and compared the two methods. We apply the proposed method to demonstrate its accuracy in estimating distance cost. In the future, this new method can be used in further surveys to provide a highly precise distance cost index. In this study, we choose Yuli as our target area is because of we found that the prevalence of diabetes and hypertension increased at a significantly higher rate in Yuli Township than in Hualien from 2017 to 2019.

In conclusion, this study was conducted because of the increasing importance of human rights and national privacy in countries around the world. In particular, personal medical records and residential addresses are highly personalized, so obtaining legal use must go through very complicated administrative procedures or obtain national consent. In Taiwan, there is Taiwan's Personal Data Protection Act to regulate the use of data, and local governments have to spend a lot of time and money to obtain the data, which is often not available in time to meet the reference for decision making in resource allocation. Therefore, we hope to develop a in time method in the present study that is derived from publicly available data, and to confirm its reliability.

## Materials and methods

**Figure 1:**
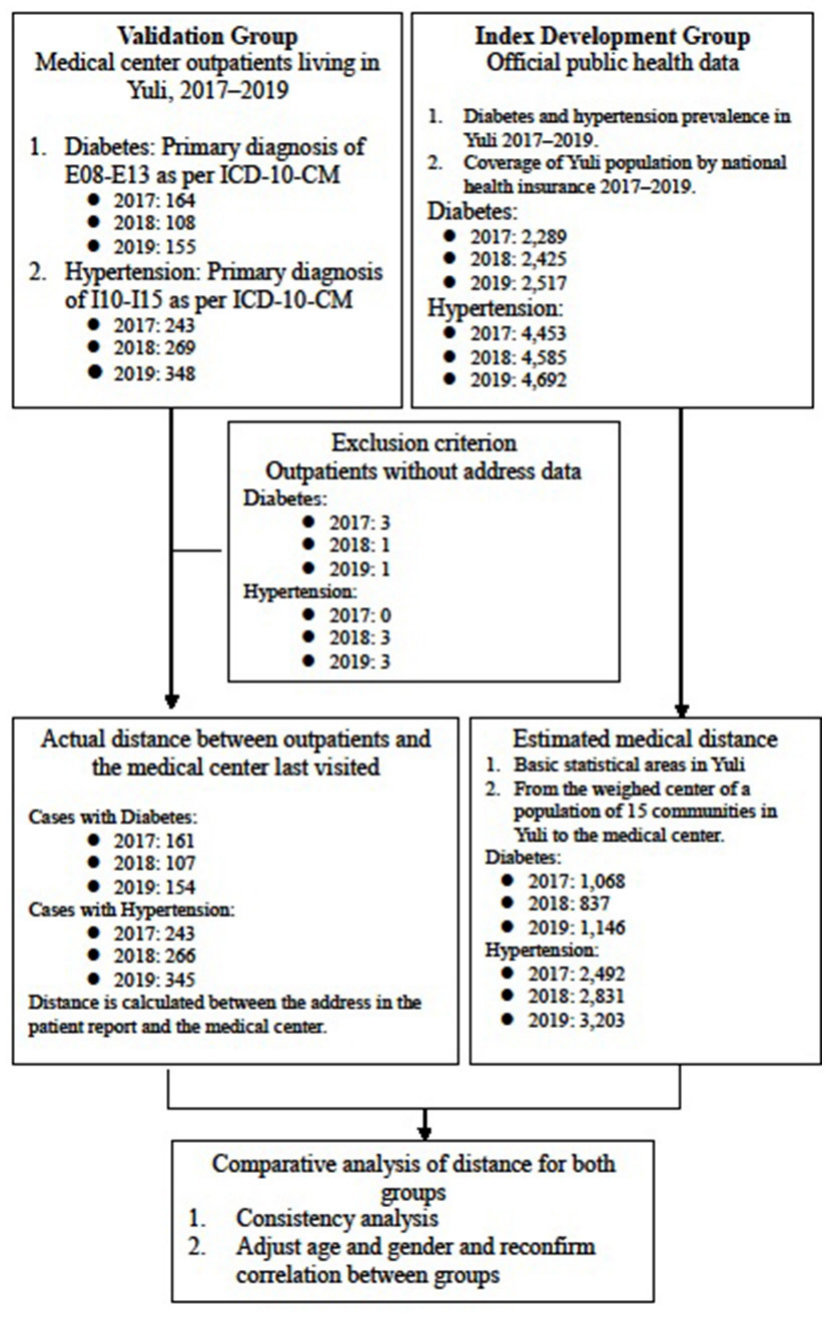
Study framework.

Figure 1 illustrates the framework of this cross-sectional study. We compared the medical distances recorded by the novel index development group and a validation group. For the index development group, we estimated the distance cost using 2017–2019 data on diabetes and hypertension prevalence in Hualien County and the number of patients in the county's Yuli Township. In addition, we use official public data from Taiwan's National Statistics. For the validation group, we obtained the 2017–2019 data for patients with diabetes and hypertension from one medical center in Hualien City and used the real data to calculate the average medical distance.

We conducted a multiple regression analysis and used intra-group correlation coefficients (ICC) to compare the average distance from the weighted center of the population in every statistical area to Tzu Chi Hospital in Hualien. Hualien Tzu Chi Hospital is the only medical center in Hualien County, also in eastern Taiwan which is located in north Hualien city and is responsible for many critical care tasks in eastern Taiwan. Trial registration: This study was approved by the Research Ethics Committee of the Hualien Tzu Chi Hospital, Buddhist Tzu Chi Medical Foundation (IRB109-239-B).

### Methods, participant characteristics and variable/concept definitions

#### Specific nominal definitions

**Figure 2:**
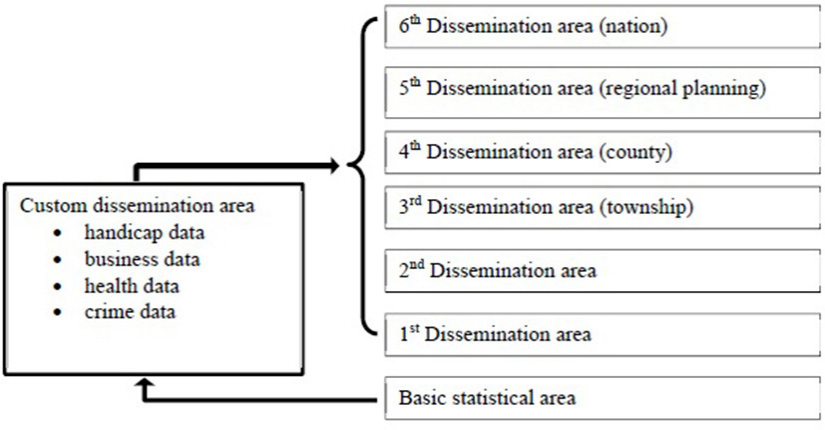
Geographical statistical classification of spatial units as per [20].

1. As per the ICD-10-CM, we defined diabetes mellitus as categories E08–E13 specified in the first three codes as a primary diagnosis in the outpatient prescription and treatment details.
2. As per the ICD-10-CM, we defined hypertension as categories I10–I15 in the first three codes of the outpatient prescription and treatment details.
3. The Statistical Office of the Ministry of the Interior builds different release area concepts based on cumulative synthesis, the minimum statistical area is the basic spatial unit for data collection, factors such as area, house number, and population (150 to 450 people) are used as the standard for measuring the level of detail, which is much more detailed than the data in the past that used townships and urban areas as the statistical unit. Figure 2 shows the various levels of spatial units. Basic statistical areas include communities, villages, and other smaller spaces. Towns are generally considered the first level of dissemination area. Cities, counties, and other administrative areas are categorized as second or higher levels of dissemination areas. This study focuses on communities as a spatial unit and accordingly, estimates the number of basic statistical areas in a community using data from the National Development Council (NDC) (22).

#### Patient and public involvement: Patient and public involvement. The secondly data was used in this study

##### Index development group

For this group, we used official public data published 2017–2019 by the National Health Insurance Administration, Ministry of Health and Welfare and National Development Council. The data include population numbers and the prevalence of diabetes and hypertension in Yuli. There are “insurance population,” “Living population” and “Prevalence” of diabetes and hypertensive that information are from Publicly Available Government Demographics and National Health Insurance Administration Ministry of Health and Welfare in Table 1. We used Living population ^*^ Diabetes and Hypertension Prevalence (official public data from National Health Insurance Administration Ministry of Health and Welfare) to get Estimated patient. Because the above 95% Living population is Insurance population that is high coverage, so we used it as insurance population to estimate the patients.

**Table 1:** The prevalence of diabetes and hypertensive from official public data in Yuli.

**Years**	**Diabetes**				**Hypertensive**			
	**Insurance population**	**Living population^1^**	**Prevalence^2^ (%)**	**Estimated patient^3^**	**Insurance population**	**Living population^1^**	**Prevalence ^2^ (%)**	**Estimated patient^3^**
2017	23,643	23,725	9.65	2,289	23,643	23,725	18.77	4,453
2018	23,244	23,725	10.22	2,425	23,244	23,725	19.33	4,585
2019	23,008	23,725	10.61	2,517	23,008	23,725	19.78	4,692

3 = 1*2: “Living population * Diabetes and Hypertension Prevalence (official public data from National Health Insurance Administration Ministry of Health and Welfare) = Estimated patient.

Because the above 95% Living population is Insurance population that is high coverage, so we used it to estimate the patients.

We estimated the number of persons with diabetes as 2,289, 2,425 and 2,517 and that of persons with hypertension as 4,453, 4,585, and 4,692 in 2017, 2018 and 2019, respectively (Table 1).

The disease prevalence is in order to estimate the patient number. We assume the total cost is the sum of the ***patient's number*** (n)^*^visit ***times*** ^*^and ***the distance from their home to the hospital***. So in the further, when we can't get the real numbers of patients and their real addresses due to patient privacy, we can use the disease prevalence and total population to estimate the possible patient group in the area from public data.

##### Validation group

We obtained data on patients with diabetes and hypertension from outpatient records gathered 2017–2019 at the only medical center in Hualien County. We estimated the number of patients with diabetes as 4,294, 4,397 and 4,994 for each year, respectively. Patients who lived outside of Yuli or who did not have their address recorded were excluded. The final sample comprised 161, 107 and 154 patients. For hypertension, we estimated 10,141, 10,521 and 10,786 persons in 2017, 2018 and 2019, respectively. After excluding cases outside of Yuli and with missing address data, the final sample consisted of 243, 266 and 345 patients with hypertension (Table 2).

**Table 2:** Characteristics of actual cases of medical center in our study: Verification group.

**Year**	**Diabetes**				**Hypertensive**			
	***N***	**Age Mean ±SD**	**Gender**		***N***	**Age Mean ±SD**	**Gender**	

			**Male (%)**	**Female (%)**			**Male (%)**	**Female (%)**
2017	161	64.16 ± 12.61	79 (48.17)	85 (51.83)	243	65.75 ± 14.84	104 (42.80)	139 (57.20)
2018	107	66.79 ± 11.41	58 (54.21)	49 (45.79)	266	67.00 ± 13.38	136 (51.12)	130 (48.88)
2019	154	64.54 ± 12.02	77 (50.00)	77 (50.00)	345	66.06 ± 13.87	168 (48.7)	177 (52.3)

### Data analysis

This study performed data collation and statistical analyses using QGIS 3.6, SAS statistical software 9.4 and SPSS Statistics 21.0. Statistical significance was considered at 0.05 for all tests. ArcGIS was used to visualize the data.

#### Spatial analysis

We conducted a spatial analysis to estimate and compare medical distance for the two groups:

1. For the index development group, we used official data on the populations in the basic statistical areas published by National Internal Affairs, Ministry of the Interior, to estimate the number of diabetes and hypertension cases in Yuli. Next, we calculated medical distance by measuring the actual distance from the center for each statistical area according to the road network. Finally, we used QGIS to establish an origin-destination matrix to analyze the distance cost of medical care from each statistical area to the hospital.
2. For the validation group, we calculated medical distance based on the outpatient addresses cited in medical records with focus on those living in Yuli's basic statistical areas. D is total distance is estimated as $D=\overset{n}{\underset{ij=1}{\sum }}dij$, where n is the patients' number, *ij* is means location, *d*_*ij*_ is the distance between those living in *ij* location and the medical center (Tzu Chi hospital).

**Figure 3:**
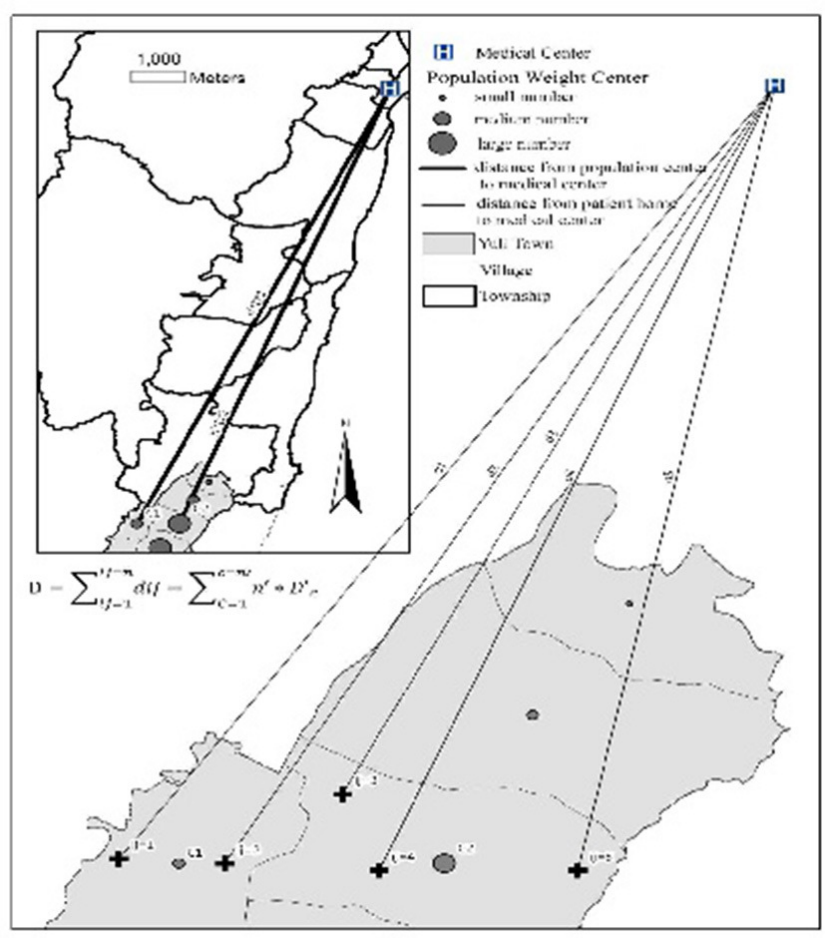
Graphical representation of the spatial analysis. D: is total distance is estimated; N: is the patient's number; *ij*: patients' real live location; *d*_*ij*_: is the distance between those living in *ij* location and the medical center (Tzu Chi hospital). c: the population center location; D'c: the distance between the center of the population and the medical center (Tzu Chi hospital).

We attempted to prove $D=\overset{ij=n}{\underset{ij=1}{\sum }}dij={\overset{c=n^{\prime }}{\underset{C=1}{\sum }}n^{\prime }*D^{\prime }}_{c}$, where *d*_*ij*_ is the distance between those living in *ij* location and the medical center (Tzu Chi hospital) (validation group) and ${D^{\prime }}_{c}$ is the distance between the center of the population and the medical center (Tzu Chi hospital) (index development group) (Figure 3).

Symbolic interpretation:

- D: is total distance is estimated
- N: is the patients' number,
- *ij:* patients' real live location
- *d*_*ij*_: is the distance between those living in *ij* location and the medical center (Tzu Chi hospital).
- *c*: the population center location
- ${D^{\prime }}_{c}:\;$The distance between the center of the population and the medical center (Tzu Chi hospital).

#### Statistical methods

Percentage, mean and standard deviation were used to describe the data. We conducted a consistency analysis and used ICC to compare the distance cost between the two groups. Finally, we performed a multiple regression analysis while adjusting for age and gender to reconfirm the correlation between the two groups.

## Results

### Participant characteristics and potential population with diabetes and hypertension

Table 1 shows that the number of residents, obtained from demographic data by the Ministry of the Interior, was higher than the number of insured individuals. We used the resident population and the prevalence of diabetes and hypertension to estimate the potential patients in Yuli. The results revealed 2,289, 2,425 and 2,517 potential patients with diabetes and 4,453, 4,585 and 4,692 potential patients with hypertension in 2017, 2018 and 2019, respectively. Age and gender data for the population with diabetes, hypertension or any other disease are not publicly available.

Table 2 presents the age and gender for outpatients of the medical center for 2017–2019. The average age of patients with diabetes was between 64.16 and 66.79 years and the gender ratio was about 1. For patients with hypertension, the mean age was 65.75–67.0 years and the rates of female outpatients were 57.2, 48.88, and 52.3% in 2017, 2018 and 2019, respectively.

Table 3 presents data obtained from the Nutrition and Health Survey in Taiwan (23). We found that the prevalence of diabetes and hypertension increased at a significantly higher rate in Yuli Township than in Hualien from 2017 to 2019. The Nutrition and Health Survey shows that the prevalence of diabetes and hypertension during 2015–2018 in Taiwan overall was higher than the numbers recorded in Hualien and Yuli because the survey only included adults over 18 years old.

**Table 3:** Descriptive statistics of the prevalence rates of diabetes and hypertension in Hualian and Yuli 2017–2019.

**Year**	**County**	**Hualien**		**Yuli**		**Taiwan** ^**a**^	
	**Disease**	**Diabetes**	**Hypertensive**	**Diabetes**	**Hypertensive**	**Diabetes**	**Hypertensive**
2017	N	318,449	318,449	23,643	23,643	9–11%	25.06%
	Prevalence (%)	7.07%	11.70%	9.65%	18.77		
2018	N	317,646	317,646	23,244	23,244		
	Prevalence (%)	7.02%	12.00%	10.22%	19.33		
2019	N	316,559	316,559	23,008	23,008		
	Prevalence (%)	7.29%	12.20%	10.61	19.78		

a The diabetes and hypertension prevalence of adults (≥18 years old) in Taiwan during 2015-2018 in NAHSIT (23).

### Medical distance cost for both groups

Yuli Township has a total of 15 communities. For each community, Tables 4, 5 show the number of basic statistical areas, medical distance and number of diabetes and hypertension cases for both the validation and index development groups. We list the distance calculated by the real address of the same community (The validation group), and the results calculated by the estimation method developed in this study (The index development group), a comparison of these 3 years. The National Internal Affairs' open data platform lists 512 basic statistical areas. Among these, we found that potential patients with diabetes in both groups lived in 109, 84 and 110 areas in 2017, 2018 and 2019, respectively. The basic statistical areas in this study account for 21.3, 16.4, and 21.5% of the total number of areas. The average distance is estimated as the medical distance between the weighted center of population in each basic statistical area (our communities) and the medical center (Tzu Chi Hospital). The concept of basic statistical area have mentioned in Method Section.

**Table 4:** The actual and estimated distances between living in 15 communities of Yuli and medical center among patients with diabetes.

**15 Communities**	**The total basic statistical area in the community (*n*)**	**Estimated case with Diabetes**			**2017**					**2018**					**2019**				
					**The basic statistical area (*n*) (%)**	**The validation group**		**The index development group**		**The basic statistical area (*n*) (%)**	**The validation group**		**The index development group**		**The basic statistical area (*n*) (%)**	**The validation group**		**The index development group**	
		**2017**	**2018**	**2019**		**Case (*n*)**	**Average distance (m)**	**Case (*n*)**	**Average distance (m)**		**Case (*n*)**	**Average distance (m)**	**Case (*n*)**	**Average distance (m)**		**Case (*n*)**	**Average distance (m)**	**Case (*n*)**	**Average distance (m)**
Sanmin	51	94	99	103	4 (7.8)	4	76,359.57	24	76,281.24	3 (5.9)	3	77,238.89	14	77,091.61	4 (7.8)	4	75,866.52	23	75,866.52
Dayu	45	115	121	126	7 (15.6)	7	80,707.09	54	81,043.37	3 (6.7)	3	80,762.11	36	81,327.22	7 (15.6)	11	80,733.18	51	80,679.82
Zhongcheng.	76	598	634	658	32 (42.1)	54	85,647.58	346	85,679.41	25 (32.9)	39	85,619.84	279	85,648.10	30 (39.5)	47	85,692.95	367	85,702.83
Yongchang	19	141	150	156	9 (47.4)	17	84,182.87	93	84,181.27	4 (21.1)	4	83,984.65	38	84,077.72	4 (21.1)	6	84,191.34	45	84,171.59
Dongfeng	21	59	63	65	1 (4.8)	1	84,135.73	9	83,903.11	2 (9.5)	2	84,239.83	26	84,460.00	2 (9.5)	2	82,824.57	15	82,824.58
Songpu	35	168	178	185	7 (20.0)	9	74,186.73	64	74,900.54	6 (17.1)	7	75,237.49	52	75,512.68	8 (22.9)	9	73,998.18	81	74,006.44
Changliang	50	74	78	81	2 (4.0)	2	92,068.66	21	91,973.45	3 (6.0)	3	92,289.67	24	92,863.08	1 (2.0)	2	92,548.62	17	92,548.62
Chunri.	29	95	101	105	4 (13.8)	5	70,932.30	37	71,291.21	3 (10.3)	3	71,243.48	16	71,586.14	6 (20.7)	7	70,542.99	32	70,617.02
Taichang.	19	151	160	166	3 (15.8)	3	85,147.54	52	85,025.07	5 (26.3)	5	85,134.77	43	85,167.23	9 (47.4)	11	85,041.03	116	85,013.49
Qimo	26	181	192	199	9 (34.6)	15	84,557.03	97	84,564.63	6 23.1)	7	84,579.00	75	84,584.97	6 (23.1)	8	84,526.16	75	84,519.2
Guowu.	18	137	145	150	11 (61.1)	16	84,994.45	113	85,029.53	5 (27.8)	10	85,081.22	50	85,047.53	10 (55.6)	18	85,030.38	111	85,027.91
Yuancheng	49	128	135	140	9 (18.4)	12	87,436.29	78	87,400.96	5 (10.2)	5	87,484.84	59	87,724.74	4 (8.2)	5	87,593.85	39	87,654.48
Dewu	19	84	89	92	4 (21.1)	8	68,741.32	36	68,752.27	4 (21.1)	5	68,728.55	47	68,749.14	5 (26.3)	8	70,798.78	44	71,949.99
Lehe	24	104	110	114	5 (20.8)	5	89,740.39	34	89,510.18	2 (8.3)	2	92,705.79	21	91,939.76	6 (25.0)	8	89,385.27	59	89,671.42
Guanyin	31	160	169	175	2 (6.5)	3	78,639.23	11	83,401.98	8 (25.8)	9	79,372.56	57	80,302.28	8 (25.8)	8	79,372.00	70	79,372.00
Total	512	2289	2425	2517	109 (21.3)	161	83,134.01	1,068	83,271.32	84 (16.4)	107	83,030.16	837	83,178.27	110 (21.5)	154	82,671.46	1,146	82,325.78

**Table 5:** The actual and estimated distances between living in 15 communities of Yuli and medical center among patients with hypertensive.

**15 Communities**	**The total basic statistical area in the community (*n*)**	**Estimated case with hypertension**			**2017**					**2018**					**2019**				
					**The basic statistical area (*n*) (%)**	**The validation group**		**The index development group**		**The basic statistical area (*n*) (%)**	**The validation group**		**The index development group**		**The basic statistical area (*n*) (%)**	**The validation group**		**The index development group**	
		**2017**	**2018**	**2019**		**Case (*n*)**	**Average distance (m)**	**Case (*n*)**	**Average distance (m)**		**Case (*n*)**	**Average distance (m)**	**Case (*n*)**	**Average distance (m)**		**Case (*n*)**	**Average distance (m)**	**Case (*n*)**	**Average distance (m)**
Sanmin Vil.	51	183	188	192	10 (19.6)	13	75,336.14	99	75,778.59	8 (15.7)	12	75,881.81	96	75,827.93	15 (29.4)	28	75,377.10	147	75,567.42
Dayu Vil.	45	223	230	235	6 (13.3)	11	80,921.94	112	80,796.46	4 (8.9)	10	80,857.65	112	81,012.95	11 (24.4)	24	81,200.83	169	81,088.30
Zhongcheng Vil.	76	1,164	1,198	1,226	39 (51.3)	76	85,571.86	734	85,615.90	43 (56.6)	75	85,636.27	805	85,642.58	45 (59.2)	93	85,631.25	887	85,672.39
Yongchang Vil.	19	275	283	290	7 (36.8)	12	84,159.11	147	84,167.87	9 (47.4)	15	84,041.04	188	84,060.80	12 (63.2)	29	84,106.45	238	84,137.03
Dongfeng Vil.	21	115	119	121	2 (9.5)	2	84,778.18	23	84,438.98	3 (14.3)	4	85,233.40	70	84,790.41	5 (23.8)	7	84,256.75	97	84,174.53
Songpu Vil.	35	328	337	345	13 (37.1)	19	74,201.09	210	74,507.30	12 (34.3)	24	74,330.53	214	74,563.56	17 (48.6)	28	74,506.62	275	74,582.81
Changliang Vil.	50	144	148	152	3 (6.0)	4	91,050.87	15	91,116.34	5 (10.0)	6	92,076.47	56	92,101.98	6 (12.0)	8	92,194.01	81	92,162.57
Chunri Vil.	29	186	191	196	5 (17.2)	8	70,670.29	58	70,813.51	6 (20.7)	8	70,958.69	59	70,894.40	8 (27.6)	8	70,504.19	89	70,774.91
Taichang Vil.	19	294	303	310	12 (63.2)	18	84,947.77	262	84,986.77	9 (47.4)	17	85,028.50	213	85,053.52	8 (42.1)	20	85,167.30	204	85,101.73
Qimo Vil.	26	352	363	371	11 (42.3)	16	84,530.86	214	84,509.53	12 (46.2)	19	84,518.85	221	84,559.36	14 (53.8)	18	84,554.44	273	84,560.39
Guowu Vil.	18	266	274	281	10 (55.6)	23	85,094.05	185	85,219.37	12 (66.7)	25	85,016.98	233	85,014.42	12 (66.7)	28	84,994.38	227	85,013.08
Yuancheng Vil.	49	249	256	262	5 (10.2)	10	86,871.67	118	87,274.66	9 (18.4)	18	87,035.71	174	87,355.51	10 (20.4)	19	86,979.71	159	87,531.92
Dewu Vil.	19	163	168	171	6 (31.6)	6	68,805.85	87	69,216.56	7 (36.8)	8	68,482.54	116	71,887.97	7 (36.8)	11	68,607.77	119	71,887.97
Lehe Vil.	24	202	208	213	8 (33.3)	11	88,726.06	114	90,036.39	9 (37.5)	16	89,262.79	129	90,282.04	9 (37.5)	15	90,485.56	149	90,744.42
Guanyin Vil.	31	310	320	327	7 (22.6)	14	79,127.97	114	79,647.87	9 (29.0)	9	79,519.14	144	79,848.78	6 (19.4)	9	79,446.87	91	80,445.28
Total	512	4,453	4,585	4,692	144 (28.1)	243	82,699.06	2492	82,408.25	157 (30.7)	266	83,017.43	2,831	82,967.03	185 (36.1)	345	82,679.75	3,203	82,492.23

For the validation group, the Changliang community reported the longest medical distance to the medical center (92,068.66, 92,289.67, and 92,548.62 m), while the Dewu community showed the shortest distance (68,741.32, 68,728.55, and 70,798.78 m). Similarly, for the index development group, the Changliang (91,973.45, 92,863.08, and 92,548.62 m) and Dewu communities (68,752.27, 68,749.14, and 71,949.99 m) reported the longest and shortest distance.

The findings were similar for potential patients with hypertension. For the validation group, the Changliang and Dewu communities reported the longest and shortest medical distances. The longest distances were 91,050.87, 92,076.47, and 92,194.01 m and the shortest were 68,805.85, 68,482.54, and 68,607.77 m in 2017, 2018, and 2019, respectively. For the index development group, Changliang showed the longest distance (91,116.34, 92,101.98, and 92,162.57 m), while we observed the shortest distance for the Dewu community in 2017 (69,216.56 m) and 2019 (68,607.77 m) and for Chunri Vil in 2018 (70,894.40 m). The basic statistical areas chosen for this study account for 28.1, 30.7, and 36.1% of the 512 basic statistical areas during the three-year research period.

### Accuracy of estimated medical distance

We used ICC to evaluate the accuracy of the estimated medical distance for the index development group. Table 6 shows the correlation of medical distance between the validation and index development groups for the cases of diabetes. Further, irrespective of whether we used the basic statistical areas or the 15 communities as the weighted center of population, we found high correlations between the two groups for all 3 years. ICC was >0.98 (p < 0.001) for the diabetes cases. Similarly, for the hypertension cases (Table 7), we observed a high correlation between the two groups (ICC: 0.96–0.99, *p* < 0.001).

**Table 6:** Predictive accuracy of diabetes (ICC).

**Years**	Group^*^	**Patient**	**The basic statistical area (512 area)**							**Community (15 communities)**						
			**The basic statistical area (n)**	**Patient**	**Average distance (m)**	**ICC**	**95% Confidence interval**		***p*-value**	**The basic statistical area**	**Patient**	**Average distance (m)**	**ICC**	**95% Confidence interval**		***p*-value**
							**Upper**	**Lower**						**Upper**	**Lower**	
2017	Group 1	161	109	161	83,134.01	0.992	0.994	0.989	< 0.001	15	161	83,134.01	0.987	0.996	0.963	< 0.001
	Group 2	2,289	109	1,068	83,271.32					15	1,068	83,235.39				
2018	Group 1	107	84	107	83,030.16	0.988	0.992	0.983	< 0.001	15	107	83,030.16	0.983	0.994	0.951	< 0.001
	Group 2	2,425	84	837	83,178.27					15	837	83,178.27				
2019	Group 1	154	110	154	82,671.46	0.984	0.988	0.978	< 0.001	15	154	82,671.46	0.989	0.996	0.966	< 0.001
	Group 2	2,517	110	1,146	82,325.78					15	1,146	82,624.01				

* Group 1: Verification group; group 2: Index development group.

**Table 7:** Predictive accuracy of hypertensive (ICC).

**Years**	Group^*^	**Patient**	**The basic statistical area (512 Area)**							**Community (15 communities)**						
			**The basic statistical area (n)**	**Patient**	**Average distance (m)**	**ICC**	**95% Confidence interval**		***p*-value**	**The basic statistical area (n)**	**Patient**	**Average distance (m)**	**ICC**	**95% Confidence interval**		***p*-value**
							**Upper**	**Lower**						**Upper**	**Lower**	
2017	Group 1	243	144	243	82,699.06	0.997	0.998	0.996	< 0.001	15	243	82,699.06	0.994	0.998	0.983	< 0.001
	Group 2	4,453	144	2,492	82,408.25					15	2,492	82,408.25				
2018	Group 1	266	157	266	83,017.43	0.959	0.968	0.948	< 0.001	15	266	83,017.43	0.993	0.998	0.980	< 0.001
	Group2	4,585	157	2,831	82,967.03					15	2,831	82,967.03				
2019	Group 1	345	185	345	82,679.75	0.966	0.973	0.959	< 0.001	15	345	82,679.75	0.997	0.999	0.992	< 0.001
	Group 2	4,692	185	3,203	82,492.23					15	3,203	82,492.23				

* Group 1: Verification group; group 2: Index development group.

We also performed a multiple regression analysis on the validation groups for diabetes and hypertension (Tables 8, 9). After adjusting for age and gender, the medical distance for the validation group continued to report a significant correlation with that for the index development groups (*p* < 0.001). The adjusted β were all >0.95 for 2017, 2018, and 2019 (adjusted R^2^ = 0.95). All regression analysis outcomes were obtained using the following formula:

**Table 8:** Predictive accuracy of diabetes (multiple regression analysis).

**Years**	**Variables**	**The basic statistical area**				
		**β**	**Adjusted β**	**95% Confidence interval**		***p*-value**
				**Upper**	**Lower**	
2017	Constant	2,074.109		3,807.569	340.649	0.019
	The distance of the group^a^	0.974	0.994	0.994	0.954	0.000
	Gender^b^	151.870	0.015	362.044	−58.305	0.155
	Age	1.867	0.005	10.111	−6.377	0.655
	Adjusted R^2^ = 0.984
2018	Constant	2,595.541		5,359.866	−168.784	0.065
	The distance of the group^a^	0.972	0.988	1.002	0.943	0.000
	Gender^b^	195.622	0.018	522.617	−131.374	0.238
	Age	−3.651	−0.008	10.793	−18.095	0.617
	Adjusted R^2^ = 0.977
2019	Constant	2,773.522		5,329.766	217.279	0.034
	The distance of the group^a^	0.968	0.985	0.996	0.939	0.000
	Gender^b^	65.022	0.006	383.084	−253.039	0.687
	Age	1.275	0.003	14.393	−11.843	0.848
	Adjusted R^2^ = 0.968

The dependent variable: the medical distance of Index development group.

a Verification group.

b Gender: male:1, female: 0.

**Table 9:** Predictive accuracy of hypertension (multiple regression analysis).

**Years**	**Variables**	**The basic statistical area**				
		**β**	**Adjusted β**	**95% Confidence interval**		***p*-value**
				**Upper**	**Lower**	
2017	Constant	1,049.248		1,894.287	204.209	0.015
	The distance of the group^a^	0.988	0.996	0.998	0.979	0.000
	Gender^b^	67.061	0.006	169.947	−35.824	0.200
	Age	0.143	0.000	3.539	−3.252	0.934
	Adjusted R^2^=0.994
2018	Constant	4,573.560		7,623.760	1,523.359	0.003
	The distance of the group^a^	0.941	0.958	0.975	0.907	0.000
	Gender^b^	273.044	0.025	657.046	−110.959	0.163
	Age	5.390	0.013	19.740	−8.960	0.460
	Adjusted R^2^ = 0.92
2019	Constant	3,850.174		6,218.332	1,482.015	0.002
	The distance of the group^a^	0.954	0.966	0.981	0.927	0.000
	Gender^b^	196.362	0.018	495.525	−102.801	0.198
	Age	0.745	0.002	11.538	−10.047	0.892
	Adjusted R^2^ = 0.92

The dependent variable: the medical distance of Index development group.

a Verification group.

b Gender: male:1, female: 0.

Diabetes cases in 2017 (Table 8).

**Estimated medical distance** **=** **0.994** ^*^medical distance of validation group **+** **0.015** ^*^ **gender** **+** **0.005** ^*^ **age (year)**

Gender: male = 1, female = 0

Gender and age are not statistically significant (*p* > 0.05)

Adjusted R^2^ = 0.984

As shown visually in Figure 3, we established the following on the basis of ICC and the regression analysis results:

$D=\overset{ij=n}{\underset{ij=1}{\sum }}dij={\overset{c=n^{\prime }}{\underset{C=1}{\sum }}n^{\prime }*D^{\prime }}_{c}\;$

- D: is total distance is estimated
- N: is the patients' number,
- *ij:* patients' real live location
- *d*_*ij*_: is the distance between those living in *ij* location and the medical center (Tzu Chi hospital).
- *c*: the population center location
- ${D^{\prime }}_{c}:\;$The distance between the center of the population and the medical center (Tzu Chi hospital).

## Discussion

The key contribution of this study is a high-precision approach for estimating the distance cost for medical services using population and disease prevalence data without revealing patients' real locations. More specifically, we developed an equation to calculate the distance cost that can be applied to cases of chronic disease. Our method can be used to predict medical distance cost using data on the frequency of patient visits. However, researchers must account for certain conditions and assumptions prior to applying this method to other situations.

### Types of disease applications

This study focused on diabetes mellitus and hypertension to estimate the medical distance in consideration of the prolonged nature of chronic disease. The number of chronically ill patients who are cured is lower than patients who experience acute and non-chronic diseases, trauma and accidents. Moreover, the frequency of medical treatment for patients with chronic diseases is more stable, and such patients tend to make repeated visits to a physician or hospital (24, 25). Importantly, accounting for patients with chronic diseases will help local governments estimate long-term medical costs. When the central government's budget for health care or long term care is based on morbidity and the number of elderly people, it is very unfair to the remote villages, which are usually also aging cities with few health care institutions, and the cost of distance can be used as an important factor to negotiate with the central government for an increase in subsidies or budget. An objective of Taiwan's Long-term Care Services Act is to set up long-term care services in residential areas of older adults and individuals with diabetes and hypertension to allow them to “age in place” in familiar locations (26). In order to achieve the goal of aging in place, it is important to have a more accurate and immediate estimate of the distance of access to care or services.

In addition, many studies have shown that spatial interactions between those in need of medical attention and hospitals are influenced by residential location and social demographics, including gender, race and socioeconomic factors. Age and gender are better available from government open data, other economic income remains private, and age and gender are also important risk factors for chronic diseases; for Hualien County, it consists mainly of rural townships, with elderly and aboriginal communities making up the majority of the population, so ethnicity variables would be not confounders. Therefore, we adjusted for age and gender in our regression analysis to more accurately examine the relationship between medical distance between the two groups (Tables 8, 9) (27–29).

In addition to chronic diseases, our estimator can be used to account for the distance cost in treating certain rare diseases, such as chromosomal abnormalities and autoimmune diseases including lupus erythematosus, and inhabitants with special needs who require rehabilitation medicine and early treatment.

Compared with chronic diseases and long-term care needs, non-communicable diseases also can be used to estimate the cost. But it is important to note that this estimation in the present study is due to the consideration of patient privacy, and the actual address cannot be obtained. So, we use the disease prevalence to estimate the number of patients with a specific region and use the distance from each statistical area to the hospital in this region, it should be more accurate to use a fixed area and stable diseases for application.

### Medical treatment selection and residents' healthcare seeking behaviors

Residents' healthcare seeking behaviors are an important factor influencing the proposed method of medical costs estimation. In this case, residents' behavior is dependent on the type of disease, their gender, their income and the medical resources available to residents (24, 25, 30, 31). Frequency of medical utilization, which is the number of times an individual uses a medical service at a target hospital, and medical distance, which is the distance between a patient's location and a target hospital, can be used to estimate medical costs for small regions or for one medical institution. However, if a region has multiple medical resources or if the target diseases are of a specific type, such as early stage cancer, then the estimation of medical distance may require data on the utilization of multiple medical services since patients tend to engage in “shopping” behavior to compare medical services (32). Thus, medical utilization behavior is necessary to include when attempting to accurately estimate the cost of medical distance. This study uses diabetes and hypertension to estimate the distance cost because they are chronic diseases and most of the patients tend to be treated at same hospitals, so this method can estimate the distance cost more accurately. The multiple medical utilization behavior would be not as our limitation.

### Travel time and distance

Approaches to calculate travel distance between medical service consumers and providers that use relatively linear distance as a basis overlook not only differences between travel distance and medical resource selection but also road network and obstacles present in challenging terrain, such as rivers and mountains. Therefore, it is more appropriate to calculate travel distance on the basis of the actual road network. Hualien County has two main traffic routes between Yuli and the medical center, of which one runs along a coastline and is generally used for sightseeing, while the other is a mountain road for local residents and the shortest straight-line distance. In this case, using a relatively linear distance does not affect our study results and reflects the actual conditions of Hualien. Thus, convenient transportation and multiple travel paths in a county or city are important factors to consider.

### Potential benefits of application in medical resource allocation policy

A medical resource allocation policy is critical in a country with a national health insurance system to ensure distributive justice. Taiwan's central government has traditionally depended on population rate or the ratio of the population to physicians, nurses and hospital beds to prioritize medical resource allocation and to determine resource deficiencies. There are special medical support programs for those with specific diseases or living in remote areas or outlying islands. However, these programs are supplementary and focus on sub-groups or low development areas. Some studies highlight that allocation policies should refer to a population's health needs, and these health needs are generally are based on medical utilization, outpatient and inpatient services, medication requirements, and the incidence and prevalence of diseases (15, 33). At present, local governments focus on the shortest travel time and the ratio of medical services to the population to understand medical needs. They must additionally consider medical distance and spatial distribution (34, 35). Determining the shortest path or travel time requires knowledge of the patients' addresses, which are becoming increasingly difficult to obtain from the National Health Insurance Database or other medical records.

The method developed in this study to estimate medical distance requires only official public information. Thus, it not only protects patient data but also is time efficient. Further, the central government can use the proposed method to determine more effective and reasonable resource allocation strategies.

## Conclusions

This study developed a cost indicator for medical distance using data from the National Health Insurance Database. The approach addresses limitations associated with various databases and the issue of patient privacy protection. In addition, it is not restricted to administrative regions but delves deeper into townships to avoid oversimplification of distance measurement. We also used data on the incidence of chronic diseases or the distribution of disease prevalence to estimate medical distance and found a high correlation with the actual distance patients traveled. This study has key implications for health policy planning and healthcare promotion and can be applied to enhance medical resource allocation as well as to improve the quality of regional medical care and preventive healthcare.

## Data availability statement

The raw data supporting the conclusions of this article will be made available by the authors, without undue reservation.

## Ethics statement

This study was approved by the Research Ethics Committee of the Hualien Tzu Chi Hospital, Buddhist Tzu Chi Medical Foundation (IRB109-239-B). Written informed consent for participation was not required for this study in accordance with the national legislation and the institutional requirements.

## Author contributions

C-FY was responsible for article conception, design, drafting, and data interpretation. S-JG analyzed the data and drafted the Methods Section. C-FY and H-CC contributed to the writing of the article. All authors contributed to the concept and design, data analysis and interpretation, manuscript drafting or revision, and have read and agree to the published version of the manuscript. The submitted manuscript has been approved by all authors.
